# Crosslinked Silk Fibroin/Gelatin/Hyaluronan Blends as Scaffolds for Cell-Based Tissue Engineering

**DOI:** 10.3390/molecules26113191

**Published:** 2021-05-26

**Authors:** Anongnart Duangpakdee, Chavee Laomeephol, Depicha Jindatip, Peerapat Thongnuek, Juthamas Ratanavaraporn, Siriporn Damrongsakkul

**Affiliations:** 1 Biomaterial Engineering for Medical and Health Research Unit, Faculty of Engineering, Chulalongkorn University, Bangkok 10330, Thailand; 6273028021@student.chula.ac.th (A.D.); papomchavee@gmail.com (C.L.); Peerapat.T@chula.ac.th (P.T.); Juthamas.R@chula.ac.th (J.R.); 2 Department of Chemical Engineering, Faculty of Engineering, Chulalongkorn University, Bangkok 10330, Thailand; 3 Department of Anatomy, Faculty of Medicine, Chulalongkorn University, Bangkok 10330, Thailand; depicha.j@chula.ac.th; 4 Biomedical Engineering Program, Faculty of Engineering, Chulalongkorn University, Bangkok 10330, Thailand; 5 Biomedical Engineering Research Center, Faculty of Engineering, Chulalongkorn University, Bangkok 10330, Thailand

**Keywords:** silk fibroin, gelatin, hyaluronan, scaffolds, 3D cell culture

## Abstract

3D porous scaffolds fabricated from binary and ternary blends of silk fibroin (SF), gelatin (G), and hyaluronan (HA) and crosslinked by the carbodiimide coupling reaction were developed. Water-stable scaffolds can be obtained after crosslinking, and the SFG and SFGHA samples were stable in cell culture medium up to 10 days. The presence of HA in the scaffolds with appropriate crosslinking conditions greatly enhanced the swellability. The microarchitecture of the freeze-dried scaffolds showed high porosity and interconnectivity. In particular, the pore size was significantly larger with an addition of HA. Biological activities of NIH/3T3 fibroblasts seeded on SFG and SFGHA scaffolds revealed that both scaffolds were able to support cell adhesion and proliferation of a 7-day culture. Furthermore, cell penetration into the scaffolds can be observed due to the interconnected porous structure of the scaffolds and the presence of bioactive materials which could attract the cells and support cell functions. The higher cell number was noticed in the SFGHA samples, possibly due to the HA component and the larger pore size which could improve the microenvironment for fibroblast adhesion, proliferation, and motility. The developed scaffolds from ternary blends showed potential in their application as 3D cell culture substrates in fibroblast-based tissue engineering.

## 1. Introduction

Tissue engineering is a multidisciplinary approach involving basic sciences, biomedicines, and engineering to counteract various human health problems, such as the defectiveness or loss of tissues or organs [1]. New tissues can be formed from isolated cells with an aid of biomaterial matrices, serving as an artificial extracellular matrix (ECM) for microenvironment conditioning, and bio-regulatory signals for supporting or guiding the proper bioactivities of loaded cells [2]. Hence, biomaterials play a critical role in scaffold fabrication. Types of biomaterials, as well as the preparation method can be adjusted to obtain final products with the desired features, which are suitable for selected applications [3]. Blending biopolymers is one of the strategies used to gain advantages from the additive materials as well as to overcome some limitations of each component [4].

Silk fibroin (SF) is a fibrous protein derived from cocoons of *Bombyx mori* domesticated silkworms. Apart from using silk fibers in textile manufacturing, silk fibroin is applied for several biomedical-related uses in different formats, such as fibers, membranes, particles, and 3D scaffolds, due to its outstanding mechanical performance, tunable degradability, fabrication versatility, and biocompatibility [5]. However, the limitation of SF is its bioinertness that can delay an early adhesion of cultured cells and reduce the number of growing cells [6,7,8]. An addition of inductive materials which can be recognized by cells, such as gelatin or hyaluronan, can improve the bioactivities of the final products. Gelatin (G) is a partially hydrolyzed product of collagen which possesses thermo-responsive properties. The bioactivities of gelatin result from the presence of arginine-glycine-aspartate (RGD) tripeptide, which is known as a recognitive binding site for cell-matrix interactions [9]. Hyaluronan or hyaluronic acid (HA) is a non-sulfated, anionic glycosaminoglycan which can be found in synovial fluid, vitreous humor, ECM, and loose connective tissue [9]. Various cells, especially stem cells and fibroblasts, express the receptor for HA, namely CD44, or the receptor for hyaluronan-mediated motility (RHAMM), which plays a key role in focal adhesions and cell motility [10].

Major drawbacks of G and HA are their high water solubility and low stability in physiological conditions [11]. Furthermore, regenerated SF itself can dissolve in water unless its structures turn from random coil to beta sheet or form covalent crosslinking. In our previous studies, SF/G blended materials were crosslinked using the carbodiimide reaction and their water solubility was significantly decreased [12,13]. Also, the carbodiimide reaction can be applied to crosslink polysaccharides due to the presence of carboxyl groups [14,15,16], from which covalent bridges are formed between the amines of proteins and the carboxyl groups of HA [17]. To our best knowledge, SF-based scaffolds with an incorporation of both G and HA have not been reported and systematically compared to those blended with either G or HA. In this study, therefore, SF-based scaffolds with an incorporation of G and/or HA ratio, as high as 25–50% of the scaffolding materials, were fabricated. The carbodiimide coupling reaction was used to crosslink the freeze-dried scaffolds in order for them to be employed as 3D cell culture substrates with an aim to have high swellability and support long-term cell proliferation. The crosslinking conditions were evaluated; whether the crosslinking time and the amount of reactant affected the crosslinking efficiency of the scaffolds, determined from the occupancy of the target functional groups. The developed scaffolds were then characterized for their physical characteristics and used as 3D cell culture substrates to investigate the bioactivities of cells seeded on the scaffolds. Physical properties which could influence the cellular activities were also identified to describe the findings and acknowledge the critical parameters for developing scaffolds suitable for cell-based tissue engineering.

## 2. Materials and Methods

### 2.1. Materials

Thai *Bombyx mori* silk cocoons (Nangnoi Srisaket 1) were supplied from the Queen Sirikit Sericulture Center, Nakhon Ratchasima province, Thailand. Gelatin, type A (MW = 100 kDa) was kindly provided by Nitta Gelatin Inc. (Osaka, Japan) and hyaluronan (MW = 2400 kDa) was purchased from TER Chemicals (Hamburg, Germany). Other reagents were from Sigma-Aldrich (St. Louis, MO, USA), unless otherwise stated. All reagents were of analytical grade.

Silk fibroin (SF) solution was prepared following the established protocol [18]. Briefly, the cocoons were boiled in 0.02 M Na_2_CO_3_ for 20 min to remove sericin and other soluble components, and the SF fibers were collected, repeatedly washed in deionized (DI) water, and dried in a fume hood. The SF fibers were then dissolved in 9.3 M LiBr at 60 °C for 4 h. The obtained SF solution was desalted by dialyzing against DI water for 48 h at room temperature (25 °C). The concentration of the SF solution was determined from the dry solid weight.

### 2.2. Scaffold Fabrication

**Scheme 1:**
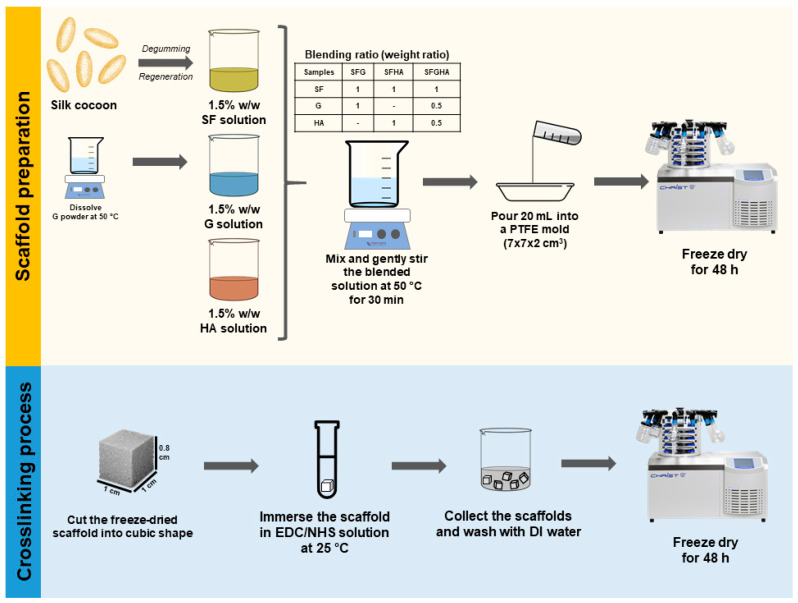
Scaffold fabrication and crosslinking process.

Scheme 1 presents the preparation process of the blended scaffolds. 1.5% *w*/*w* aqueous solutions of SF, gelatin (G), and hyaluronan (HA) were prepared and mixed in different weight ratios, namely SF:G 1:1 (SFG), SF:HA 1:1 (SFHA), and SF:G:HA 1:0.5:0.5 (SFGHA). The blended solutions were gently stirred at 50 °C for 30 min prior to pouring 20 mL of the mixtures into a polytetrafluoroethylene (PTFE) mold (7 × 7 × 2 cm^3^). The mixture concentration of 1.5% *w*/*w* was chosen because our preliminary study revealed that, at a lower concentration, very delicate scaffolds were obtained, while the mixture at a higher concentration was very viscous, resulting in non-homogeneous mixing. The blends were then frozen at −80 °C and lyophilized using a freeze-dryer (Christ^®^, Osterode am Harz, Germany). The freeze-dried scaffolds were subsequently crosslinked using the 1-ethyl-3-(3-dimethylaminopropyl)carbodiimide hydrochloride (EDC)/*N*-hydroxysuccinimide (NHS) coupling reaction. The scaffolds were cut into cubic shapes (1 × 1 × 0.8 cm^3^) and immersed in an excess volume of 80% *v*/*v* ethanolic solution (~1 mL/piece) of 20, 30 and 50 mM EDC/NHS for 6, 12, and 24 h at room temperature (25 °C), before rinsing with DI water to stop the reaction and remove redundant reactants. The crosslinked scaffolds were subsequently dried using a freeze dryer.

### 2.3. Evaluation of Crosslinking Efficiency

The effects of crosslinking time and the concentration of EDC on the characteristics of these scaffold systems, including water solubility, the content of free amines, and *N*-acetylglucosamine (NAG) groups, were investigated. The crosslinking time was trialed at 6, 12, and 24 h, and 20, 30, and 50 mM EDC was used. These characteristics could reveal the crosslinking efficiency of the EDC crosslinking reaction. To determine the water solubility of the non-crosslinked and crosslinked scaffolds, the dry weight of the scaffolds was collected after immersing in DI water for 24 h at room temperature. The remaining weight (%) was calculated.

The carbodiimide coupling reaction is used to bridge covalent bonds between amino and carboxyl groups by converting the carboxyl groups into succinimidyl esters and substituting the primary amino groups [17]. Therefore, the crosslinking can be evaluated from the reduction of the unbound amino groups. Herein, the 2,4,6-trinitrobenzene sulfonic acid (TNBS) assay was used to quantify the amount of free amines by following the established protocol [19]. In brief, the scaffolds (average weight = 5.0 ± 0.1 mg, *n* = 3) were reacted with 2 mL of 0.25% TNBS in 0.25 M NaHCO_3_ at 40 °C for 2 h, before adding 2 mL of concentrated HCl and incubating at 60 °C for 12 h to completely dissolve the scaffolds. After that, the absorbance of the solution was collected at 415 nm, and the obtained values were converted to the content of free amines using a standard curve of alanine.

NAG, the characteristic functional group of hyaluronan, was quantified using a modified Elson–Morgan assay reported by Blix [20]. The samples (average weight = 5.0 ± 0.1 mg, *n* = 3) were digested in 5 mL of concentrated HCl at 96 °C for 14 h, before neutralizing pH with 4 N NaOH. Subsequently, 2 mL of 3% *v*/*v* acetyl acetone in 1.25 N Na_2_CO_3_ was added and incubated at 96 °C for 20 min followed by the addition of Ehrlich’s reagent, which is composed of 0.18 M *p*-dimethylaminobenzaldehyde (DMAB) in 50% *v*/*v* ethanol acidified by concentrated HCl. After 60 min incubation, the absorbance at 528 nm was finally collected and the NAG amount was calculated.

### 2.4. Swellability and Degradability of the Scaffolds

The crosslinked scaffolds were cut into a cube (average weight = 5.0 ± 0.1 mg, *n* = 3), immersed in phosphate buffer saline (PBS, pH 7.4) at 37 °C for a particular time period, and collected. Weights of the samples before and after the immersion were measured and used to calculate the swellability percentage of the scaffolds.

Degradability of the scaffolds was conducted in cell culture medium (Dulbecco’s modified Eagle’s medium (DMEM; Gibco, Waltham, MA, USA) supplemented with 10% fetal bovine serum (FBS; Gibco, Waltham, MA, USA) and 1% antibiotics (Gibco, Waltham, MA, USA)) to mimic cell culture conditions. The samples (average weight = 5.0 ± 0.1 mg, *n* = 3) were incubated at 37 °C in humidified air with 5% CO_2_ for a specific period, before collecting, washing with DI water and freeze-drying. Subsequently, the weight of the remaining samples was measured and compared to their initial weight to calculate the weight loss percentage.

### 2.5. Micromorphology of the Scaffolds

The freeze-dried scaffolds were cross-sectioned by a surgical blade and sputter-coated with gold. The micromorphology was visualized using a scanning electron microscope (SEM; JSM-5410LV, Jeol, Tokyo, Japan). Pore size was determined using Image J software (20 pores per image, *n* = 3).

### 2.6. Adhesion and Proliferation of NIH/3T3 Cells on the Scaffolds

The samples were cut into a small cube (5 × 5 × 2 mm^3^), placed in non-treated containers and sterilized by ethylene oxide fumigation [21]. Mouse embryonic fibroblasts, NIH/3T3,were kindly given from Asst. Prof. Jittima Luckanagul, Faculty of Pharmaceutical Sciences, Chulalongkorn University. Cells were seeded onto the scaffolds at a density of 5 × 10^5^ cells/scaffold. Cell adhesion was determined at 2, 4 and 6 h after seeding. For the proliferation profile, cells were allowed to attach to the scaffolds for 6 h before transferring to a 6-well tissue culture treated plate (JET Biofil, Guangzhou, China). The samples were cultured in a CO_2_ incubator at 37 °C and 5% CO_2_/95% air. The number of cells were determined at each time point (1, 2, 3, 5 and 7 days) using a DNA assay.

To perform a DNA assay, the samples were washed with PBS, and a lysis buffer containing 0.02% sodium dodecyl sulfate in saline sodium citrate buffer was added to break the cells while preserving the DNA. The samples were frozen and thawed several times to ensure complete cell breakage. After that, the supernatant was collected and mixed with Hoechst 33258 (Thermo Fisher Scientific, Waltham, MA, USA) solution. The emission intensity at 460 nm was measured with an excitation wavelength of 355 nm. Cell-free scaffolds were used as blanks and the experiment was performed in quadruplicate.

Population doubling time (PDT) was calculated using the following equation:

(1) $$PDT=\frac{\ln2}{µ}$$

where µ refers to the specific growth rate, which can be determined from the slope of plots between the log number of cells in the exponential growth phase and the culture time.

### 2.7. Visualization of Cell Morphology

At a particular time, cell-loaded scaffolds were collected and fixed in cold 4% paraformaldehyde overnight. The samples were then dehydrated by ethanol gradation before drying the samples using the critical point drying apparatus (Leica EM CPD300, Leica Microsystems, Wetzlar, Germany). Micrographs of cells attached on the scaffolds were visualized by an SEM as aforementioned.

Cell morphology was also observed using an immunohistochemical analysis. The dehydrated samples were fixed in paraffin and sliced by a microtome (Leica RM2265, Leica Microsystems, Wetzlar, Germany). The fixed cells were treated with Triton X-100 to increase cell membrane permeabilization and blocked by 2% normal goat serum. Polyclonal beta-actin antibody (Thermo Fisher Scientific, Waltham, MA, USA) was added to the samples, incubated overnight, and washed with PBS, before staining with a secondary antibody, Alexa Fluor^®^ 488 goat-anti rabbit IgG (Thermo Fisher Scientific, Waltham, MA, USA). Subsequently, the samples were stained with DAPI (Abcam, Cambridge, UK). A confocal laser scanning microscope (LSM 800, Zeiss, Wetzlar, Germany) was used to visualize nuclei and actin filaments using blue and green filters, respectively.

### 2.8. Statistical Analysis

Statistics were analyzed by IBM SPSS Statistics 22 software (IBM, Armonk, NY, USA) using the one-way analysis of variance (ANOVA) with Bonferroni post-hoc tests. Significance was considered at a *p*-value ≤ 0.05.

## 3. Results

### 3.1. Crosslinking Efficiency

**Figure 1:**
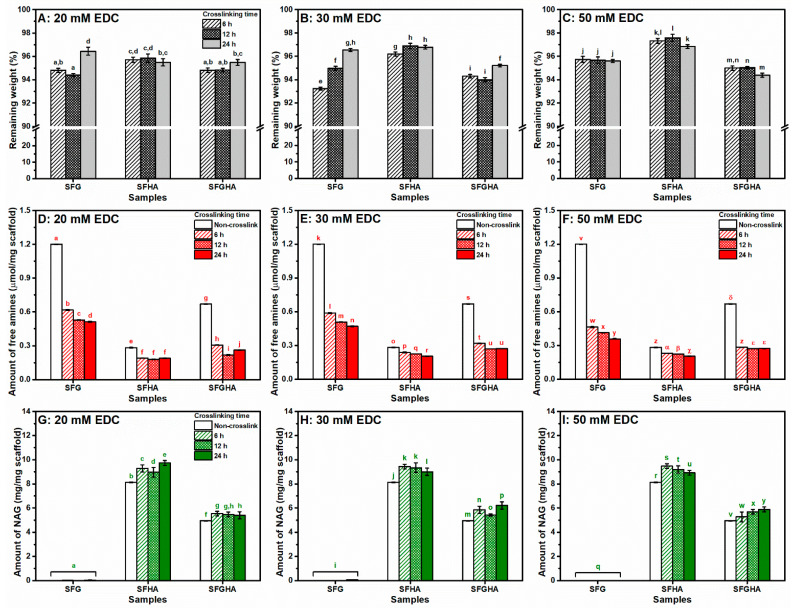
Crosslinking efficiency of the scaffolds at different EDC concentrations (20, 30, and 50 mM) and crosslinking times (6, 12, 24 h). (**A**–**C**) The remaining weight (%) of the scaffolds, (**D**–**F**) the amount of free amine groups of non-crosslinked and crosslinked scaffolds analyzed by TNBS assay, and (**G**–**I**) the amount of NAG groups determined by Elson–Morgan assay. The alphabetical letters annotate the statistical difference at a *p*-value ≤ 0.05. The statistical analysis was performed within the same concentration of EDC used in the crosslinking reaction.

Optimum conditions for crosslinking SFG, SFHA, or SFGHA blends using the EDC/NHS reaction were investigated by varying the EDC concentration and the crosslinking time. The crosslinking efficiency was determined from the changes of water solubility, and the number of free amino and NAG groups, after crosslinking. Figure 1A–C represent the remaining weight of the crosslinked scaffolds after immersion in DI water for 24 h. Before the crosslinking process, the scaffolds immediately and completely dissolved in PBS. After crosslinking, almost 95% of the scaffolds were retained even at the lowest EDC (20 mM) and the shortest reaction time (6 h), and there was no obvious relationship between the crosslinking conditions and the solubility of the scaffolds.

Since the carbodiimide reagents can form covalent bonds between primary amines and carboxyl groups [17], a reduction of the free amines after the reaction can imply the crosslinking efficiency. In this case, the number of free amines was quantified by the colorimetric TNBS assay, and the results are presented in Figure 1D–F. Comparing within the same crosslinking conditions, SFHA showed the smallest changes in the number of free amines between non-crosslinked and crosslinked scaffolds. The changes of free amines for SFG and SFGHA scaffolds were similar i.e., approximately half of free amines were consumed in crosslinking. For SFG, the crosslinking efficiency related to a longer crosslinking time, while that of SFGHA was not clearly relevant to the crosslinking conditions.

NAG groups were quantified for all non-crosslinked and crosslinked scaffolds to represent the existing HA in the scaffolds (Figure 1G–I). NAG in the SFG samples could not be measured due to the absence of HA. It seems that the amount of EDC and the reaction time showed no influence on the amount of NAG in all HA-containing scaffolds.

### 3.2. Swellability and Degradability of the Scaffolds

**Figure 2:**
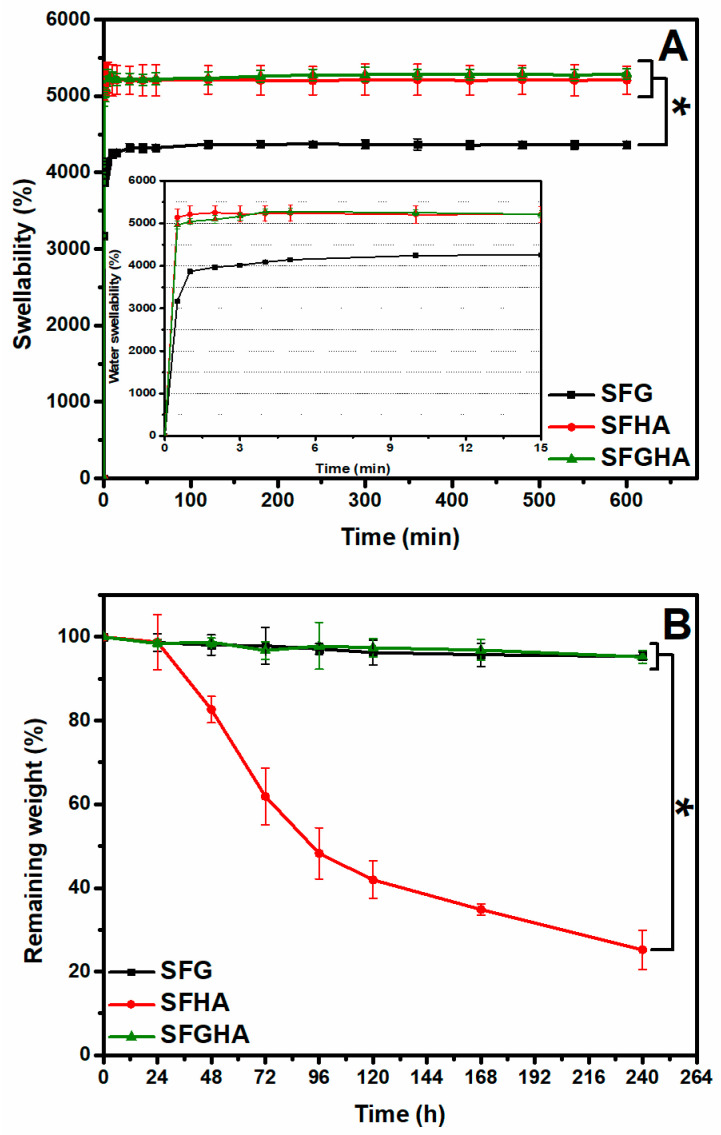
Physical properties of the crosslinked SFG, SFHA, and SFGHA scaffolds. (**A**) Swellability of the scaffolds after immersion in PBS at 37 °C. An inset presents the data in the first 15 min. (**B**) Remaining weight of the scaffolds after the incubation in cell culture medium at 37 °C. The asterisk (*) indicates the statistical difference at a *p*-value ≤ 0.05.

The physical properties of the scaffolds were evaluated to prove the applicability of the scaffolds as cell culture matrices. Figure 2A presents the swellability of the scaffolds after immersion in PBS at 37 °C. Since the non-crosslinked scaffolds completely dissolved immediately after immersion in the test media, the experiment was only performed on crosslinked samples. For all the samples, the maximum swelling percentage could be achieved within 10 min, and the scaffolds absorbed the medium to more than 40 to 50 times of their initial weight. Interestingly, HA-containing scaffolds (SFHA and SFGHA) showed a significantly higher swellability percentage than that of SFG.

Degradability of the scaffolds was conducted in the cell culture medium using the same conditions as for cell culture. It can be seen that the SFG and SFGHA retained their weight for 10 days, while the weight of SFHA significantly dropped after 24 h and gradually degraded until it reached 20% of the remaining weight after 10 days of incubation (Figure 2B).

### 3.3. Micromorphology of the Freeze-Dried Scaffolds

**Figure 3:**
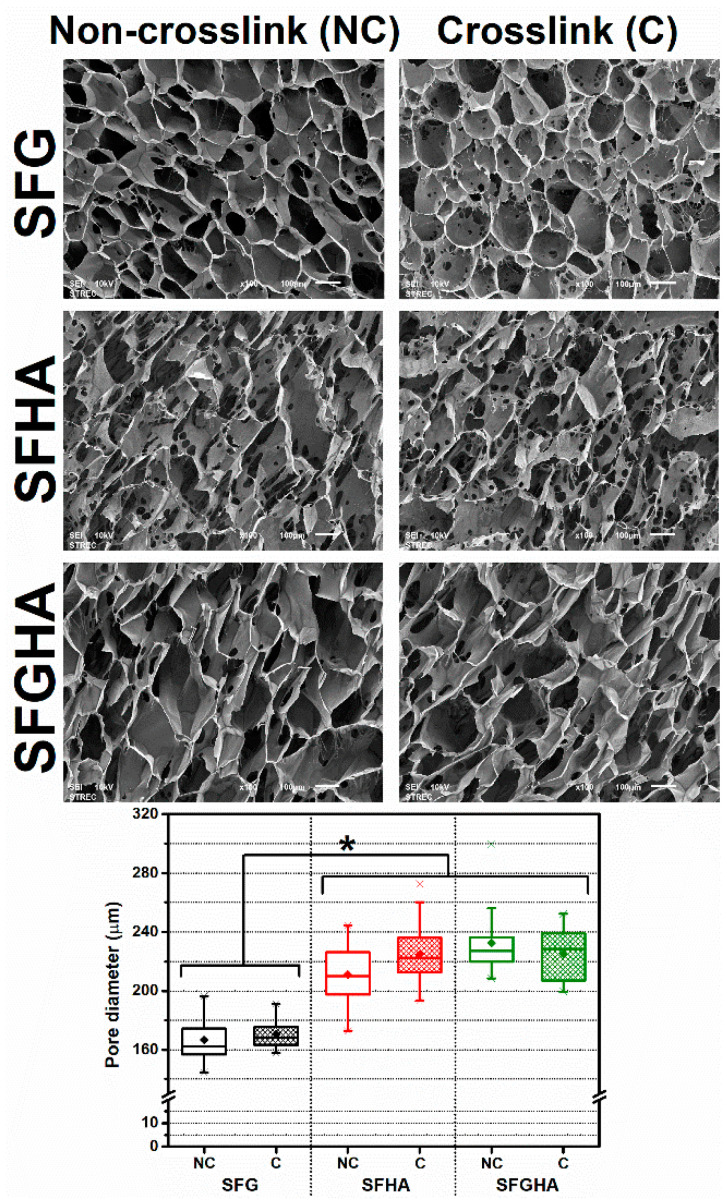
SEM micrographs of the non-crosslinked (NC) and crosslinked (C) SFG, SFHA and SFGHA scaffolds. Scale bar = 100 µm. Box plot represents pore diameter of the scaffolds evaluated from the micrographs. The asterisk (*) indicates the significance level at a *p*-value ≤ 0.05.

Figure 3 shows the SEM images and the pore diameter of the non-crosslinked and crosslinked scaffolds. For all the scaffolds, an interconnected porous structure can be observed. The small holes can be noticed throughout the wall of scaffolds, and the increased pores were noticed after crosslinking in all samples. No change in the pore diameter of each scaffold was observed after crosslinking. Comparing among the scaffolds, the pore diameter of SFG scaffolds was significantly smaller than those of SFHA and SFGHA scaffolds.

### 3.4. Cell Adhesion and Proliferation

**Figure 4:**
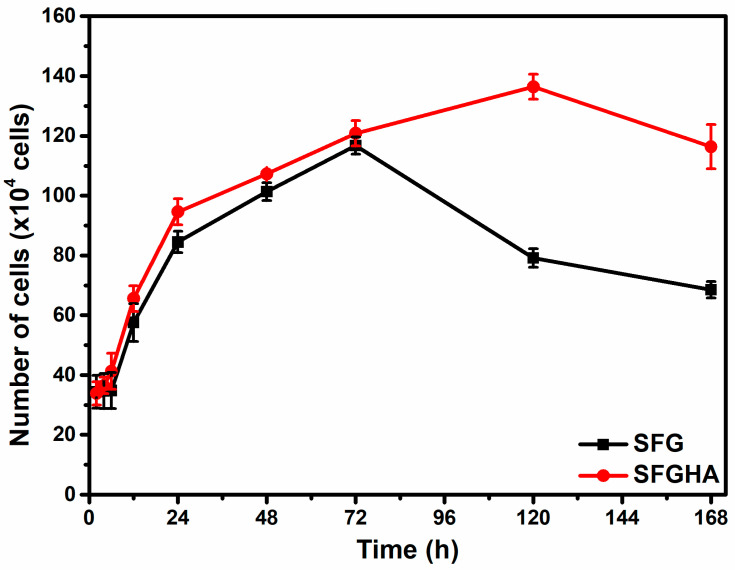
Proliferation profile of NIH/3T3 cells cultured on the SFG and SFGHA scaffolds for 7 days. The number of cells was quantified by DNA assay (*n* = 4).

Because the SFHA scaffolds degraded faster than others in the cell culture media as presented in Figure 2B, only SFG and SFGHA were tested as scaffolding. Cell attachment to the scaffolds after seeding for 2, 4, and 6 h compared to an initial seeding density of 5 × 10^5^ cells/scaffold is shown in Table 1. Approximately 70% of the cells attached to both scaffolds within the first 2 h after seeding which was not different from the number of attached cells at 4 and 6 h post-seeding. Furthermore, the results showed no statistical difference between the types of scaffolds. The cell proliferation profile of SFG revealed that the highest cell number was achieved within 72 h, before depletion afterwards. For SFGHA, the time to reach the highest cell number was prolonged to 120 h (Figure 4). However, the PDT of the cells in the exponential growth phase (2–72 h) of both groups were similar (Table 1).

### 3.5. Cell Morphology

**Figure 5:**
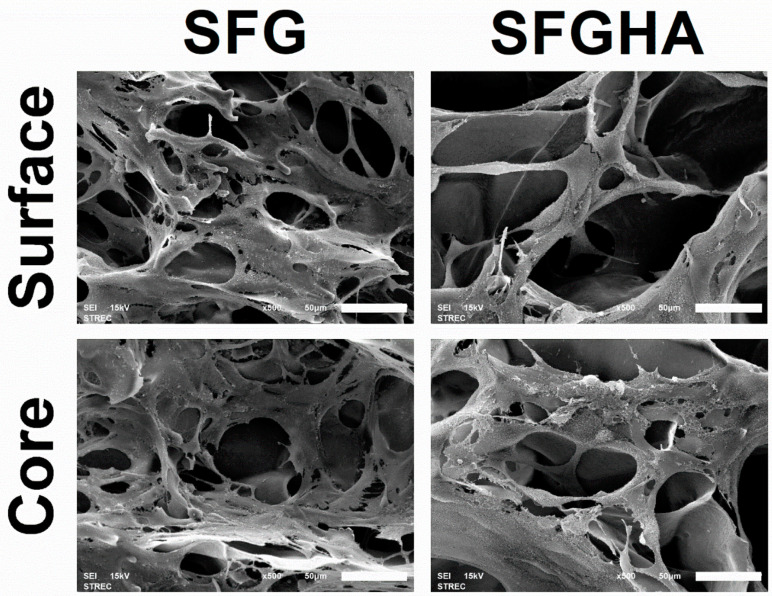
SEM micrographs of cell-loaded SFG and SFGHA scaffolds obtained at 5-day post-seeding. The images of outer surface and inner core are presented. Scale bar = 50 µm.

**Figure 6:**
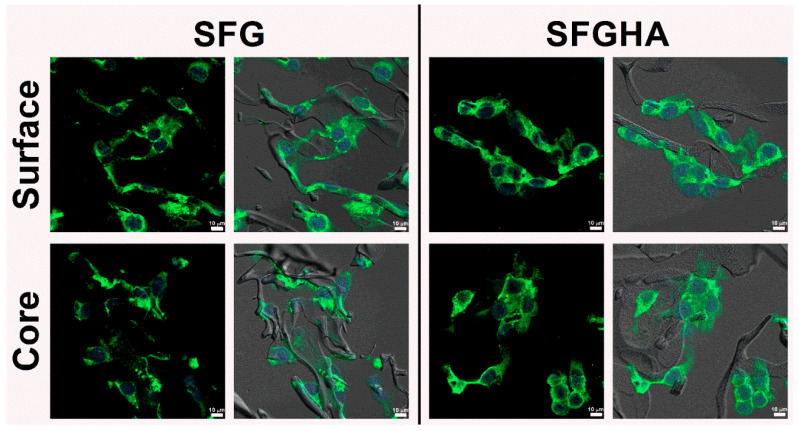
Immunohistochemical stained images of NIH/3T3-loaded scaffolds obtained after 5 days of seeding. Fluorescence images are aligned with the combined images of the bright-field and the stained images. Green and blue stains indicate the cytoplasm and nucleus, respectively. The images obtained from the scaffolds at the surface and core are shown. Scale bar = 10 µm.

After culturing the cells on the scaffolds for 5 days, the cell-loaded samples were fixed and dried, prior to micromorphology visualization by SEM, as shown in Figure 5. It can be seen that the cells were spread and attached to the wall of all scaffolds. The cross-sectioned images also showed a number of cells inside the scaffolds and their cytoskeleton was connected with the materials. The results were in accordance with the fluorescence-stained images (Figure 6). The cells were spread and aligned along the wall of scaffolds. Furthermore, the cells could migrate and grow inside the scaffolds after culturing for a particular time.

## 4. Discussion

G and HA are the biopolymers popularly used for scaffold fabrication in tissue engineering, and they are known to be unstable in several media and in physiological conditions without covalent crosslinking [11]. Also, regenerated SF can rapidly dissolve in water due to the presence of alpha-helices and random coils, but insoluble SF can be obtained after an induction of beta-sheet structure or an introduction of covalent bonds [22,23]. Table 2 presents recent publications in the last 5 years involving the fabrication of blended SF, G and HA scaffolds using various crosslinking methods. As can be seen, carbodiimides are the most studied reagents used for crosslinking between polysaccharides, such as HA, and amino-containing polymers [11]. Using EDC/NHS coupling reagents as an illustration, carboxylic groups of either polysaccharides or proteins are converted to *O*-acylisourea derivatives by EDC and form relatively stable succinimidyl esters due to a presence of NHS. These active esters can then form a complex with primary amines of lysine residues, resulting in the formation of covalent bridges between the polymer chains [17].

Crosslinking by immersing the scaffolds in the crosslinking reagents was the process used in this study to ensure no trace reactants were left in the scaffolds after washing. Furthermore, the crosslinking conditions (concentration and crosslinking time) could be controlled to achieve final products with the desired features, such as tunable mechanical properties, which could be further investigated. Compared to all of the non-crosslinked scaffolds which completely dissolved in water, the crosslinked scaffolds were significantly more stable, as they could retain their weight up to 95% when immersed in water. Crosslinking efficiency can be determined from the reduction of the available amines after crosslinking and comparing to the initial amount. Approximately 50–70% of free amines were used in the crosslinking reactions of SFG and SFGHA, while those of SFHA used about 20–30%. This result could be from the protein ratio in the scaffolds, which was 100%, 75% and 50% by weight for SFG, SFGHA, and SFHA, respectively. Since proteins offer both amines from lysine, and carboxyl groups from aspartate and glutamate, the crosslinking sites for EDC/NHS are higher than those of HA, which presents only carboxylic groups. The crosslinking efficiency of SFHA was doubtlessly lower than others due to the presence of the lowest protein amount. This is the reason why even the SFHA scaffolds were stable in culture medium for 24 h, but the long-term stability was problematic as their weight loss was up to 80% in 10 days, making them ineligible for prolonged cell culture. Our results were in accordance with previous works in which gelatin or collagen were crosslinked with HA using the EDC/NHS reaction. The results revealed that a higher content of gelatin or collagen led to a higher crosslinking degree [30,31]. Generally, the chemical crosslinking by immersing the scaffolds in the crosslinker solution can be less effective than the blending of crosslinkers with the polymer mixtures before fabrication into a scaffold. Several studies revealed that SF/HA scaffolds crosslinked by EDC/NHS while mixing in the blends before freeze-drying showed a slow degradation rate, and the stability of the samples could be retained for a longer incubation time in both buffer and enzymatic media [15,16].

The amount of NAG was determined to evaluate whether HA was lost during the crosslinking reaction, and the results showed that HA was preserved for both SFHA and SFGHA groups. The amount of NAG in SFGHA was about half of NAG in SFHA, which was reasonably related to the initial amount of HA in both scaffolds. In regard to our findings, we chose the crosslinking reaction using 20 mM EDC for 6 h, since it was enough to fabricate water-stable scaffolds.

The aims of our developed scaffolds were for serving as matrices for cell-based tissue engineering, which the scaffolds should be designed to achieve as a required feature of tissue-engineered scaffolds. SF was used as the base material to enhance the mechanical stability as well as degradability due to its characteristic physical properties [32]. However, due to the absence of cell adhesion motifs in SF, early cell adhesion can be delayed, and the number of proliferated cells can be lower than those of other bioinductive materials [6,7]. Gelatin, therefore, was introduced to the blends to enhance the biological activities of the scaffolds through the presence of cell recognition sequences, RGD motifs [11]. Furthermore, hydrophilic HA was added to the blends to improve the swellability up to 40–50 times in water, as shown in Figure 2A, which could be advantageous in producing capillary force during the cell seeding process, enhancing the cell penetration into the core of scaffolds. HA could also enhance cell-material interactions as it can be recognized by surface antigens, such as CD44, which is highly present in various types of mammalian cells, including normal cells, e.g. hematopoietic cells, epithelial cells or fibroblasts, and tumor cells [10,33]. The cell culture results revealed that an early adhesion of NIH/3T3 cells on both SFG and SFGHA was achieved within 2 h post-seeding, which could have resulted from the presence of inductive materials in the scaffolds. Compared to previous reports, the number of cells adhered to pure regenerated SF substrates was far lower than to those blended with cell inductive or chemically modified polymers [6,7].

Cell proliferation kinetics of NIH/3T3 cells cultured on SFG and SFGHA were similar, as recognized from the non-significantly different PDT (Table 1). However, the cells cultured on SFGHA achieved a higher number than those on SFG and the time to reach the highest cell number was 5 days compared to 3 days on SFG. Apart from the properties of HA in enhancing the bioactivities of fibroblasts due to the recognition of surface receptors as previously mentioned, it was possible that a larger pore size of SFGHA than that of SFG (Figure 3) could allow more space for cell proliferation. Our results were in an agreement with a previous report which stated that an addition of HA to a collagen scaffold can enhance the proliferation of human fibroblasts. The authors proposed that the presence of HA could be beneficial for microenvironments to support proper cellular activities [34].

One of the critical features for cell-based scaffolds in 3D cell culture is that the matrices should support the cells inside the scaffold to be viable and functional, which plays an important role in matrix remodeling [35]. SEM (Figure 5) and immunohistochemical staining results (Figure 6) showed that the cells were present at the core of the scaffolds after 5 days of culture, which referred that the cells could survive in an interior part of the materials. Presumably, the cells present at the core could migrate from the surface into the scaffold, which should be confirmed by a cell migration study. The introduction of the both bio-inductive materials, G and HA, in the blends can enhance cellular recognition and could support cell attachment and proliferation as well as allow cell motility from the periphery to the core to prevent local overpopulation. Additionally, porosity and interconnectivity of the scaffolds are known to allow cell proliferation and migration. There was a report confirming that scaffolds with a larger pore size allowed a higher cell migration and infiltration [36], which could describe why the higher cell number was obtained in SFGHA. Hence, our results revealed that the developed scaffolds possessed the potential to serve as biomimetic substrates for 3D cell culture.

## 5. Conclusions

In this study, the blends of SF, G, and HA at different ratios were freeze-dried to obtain the porous 3D structures and crosslinked using EDC/NHS coupling reagents. Amino groups of lysine in SF or G were covalently bridged with the carboxyl groups of HA or aspartate or glutamate of proteins. The stability of all scaffolds in water was dramatically improved after crosslinking, especially for SFGHA. However, the SFHA scaffolds showed a significant weight loss in a long-term degradability test in cell culture medium, which can be related to the insufficient crosslinking efficiency. Porosity and interconnectivity were observed in all freeze-dried samples, and the HA-containing scaffolds showed a significantly larger pore size. Though biological responses of NIH/3T3 fibroblasts showed similar cell adhesion and growth kinetics in SFG and SFGHA groups, the higher cell number was achieved in the SFGHA samples which could be caused by the presence of HA and the larger pore diameter. The presence of appropriate inductive materials in the scaffolds as well as the pore architecture could be beneficial to cell activities, including cell attachment, proliferation, and motility. Our developed scaffolds showed promise as 3D cell culture scaffolds for fibroblast-based tissue engineering. Longer cell culture times and different cells, such as primary cells or stem cells, would need to be trialed to investigate the eligibility of the scaffolds in various applications. The implantation of cell-loaded scaffolds at defect sites of an animal model could be an informative source of data to translate the uses of scaffolds towards the clinical setting.

## Figures and Tables

**Table 1:** Attachment percentage at 2, 4, and 6 h of NIH/3T3 cells cultured on the scaffolds compared to the initial seeding density at 5 × 10^5^ cells/scaffold. Specific growth rate (µ) and population doubling time (PDT) were obtained from the cells in the exponential growth period (*n* = 4).

Samples	% Attachment			µ	PDT (h)
	2 h	4 h	6 h		
**SFG**	73.87 ± 6.87	73.18 ± 11.04	74.18 ± 10.01	0.055 ± 0.008	12.88 ± 1.97
**SFGHA**	71.64 ± 3.06	75.79 ± 2.38	87.68 ± 8.58	0.050 ± 0.003	13.97 ± 0.72

**Table 2:** Comparison of the scaffolds fabricated from SF, G and HA (NA = data not available, BMSCs = bone marrow-derived stem cells, mESCs = mouse embryonic stem cells, L929 = mouse fibroblasts).

Scaffolding Materials	Crosslinking Method	Physical Properties	Biological Evaluation	Ref.
SFHA and SFHA with heparin (4%, 25% HA ratio)	EDC/NHS (blending)	Water uptake (93%), Pore size (20–140 µm), Young’s modulus (12.5–13.1 kPa)	Cytocompatible (NIH/3T3, 24 h)	[24]
SFHA (5%, 1.5-5% HA ratio)	Soft-freezing	Water solubility (4.0–4.6% with 1.5–5% HA ratio), Compressive modulus (32–57 kPa), 21-day degradation (~47% in PBS, ~72% in enzymes)	NA	[25]
SFHA (2.5% SF + 0.25% HA)	Ethanol evaporation	Swellability (20–30 times), PBS degradation (80–85% in 30 days), Compressive modulus (28–30 kPa)	SFHA scaffolds supported cell adhesion, proliferation, and migration (BMSCs, 16 days)	[26]
SFHA (2%, 5–20% HA ratio)	EDC/NHS (immersion)	Water solubility (6–8%), Swellability (5–10 times), 21-day degradation (10–12% in PBS, 75–95% in enzymes)	SFHA scaffolds supported cell adhesion, proliferation, and migration (mESCs, 9 days)	[27]
SFHA (10%, 20–40% HA ratio)	Ca^2+^-Formic acid	Water solubility (2–2.5%), Swellability (7–12 times), Compressive stress (0.01–0.2 MPa)	SFHA scaffolds supported cell adhesion and proliferation (L929, 9 days)	[28]
GHA (5%, 2% HA ratio)	EDC/NHS (blending)	Swellability (6–7 times), Elastic modulus (0.9–1 kPa), Young’s modulus (~140 kPa), Compressive modulus (~40 kPa)	GHA scaffolds supported cell proliferation (NIH/3T3, 7 days)	[29]
SFHA (2.5%, 20–60% HA ratio)	EDC/NHS (blending)	Pore size (50–100 µm), Compressive strength (3.2–11.9 kPa), 18–day degradation (6–10% in PBS, 65–90% in α-chymotrypsin)	SFHA scaffolds were biocompatible after 5-day implantation in rats	[16]
SFGHA (1.5%, 25–50% G ratio, 25–50% HA ratio)	EDC/NHS (immersion)	Water solubility (2–5%), Swellability (40–50 times), 10-day degradation (80% in culture medium), Pore size (160–240 µm)	SFG and SFGHA scaffolds supported cell adhesion, proliferation, and migration (NIH/3T3, 7 days)	This study

## Data Availability

Data sharing is not applicable to this article.

## Sample Availability

Not available.
